# Efficacy and reproducibility of response of greater occipital nerve blocks in chronic cluster headache: a large-sample prospective analysis

**DOI:** 10.1186/1129-2377-14-S1-P44

**Published:** 2013-02-21

**Authors:** N Abu Bakar, G Lambru, L Stahlhut, P Shanahan, MS Matharu

**Affiliations:** 1 Headache Group, UK

## Introduction

Greater occipital nerve block (GONB) has been shown to be an effective treatment, mainly in episodic cluster headache (CH), in two randomized double-blind placebo-controlled studies. However, not much data is available on chronic CH (CCH). In addition very little is known about the reproducibility of response to GONBs. Aim: To prospectively assess the efficacy and reproducibility of response of GONBs in a large cohort of CCH patients.

## Methods

CCH patients referred to our outpatient clinic between 2007 and 2010, and had a unilateral GONB, using a mixture of methylprednisolone 80 mg and 2 ml lidocaine 2%, were prospectively studied. Data on headache characteristics (frequency, severity and duration) were collected using headache diaries before and after the procedure. The outcomes of three subsequent GONBs performed in responders to the first, three-month apart, were also analysed.

## Results

Eighty-three CCH patients were studied. A positive response was observed in 59 (71%) patients; 42 (51%) were rendered pain free, whilst 17 (21%) had a partial benefit, lasting a median of 18 days (range: 1-504 days). There was a transient worsening of CH in 6% of patients and mild adverse effects were reported by 34%. The overall rate and average duration of response rate remained similar after the second (n = 43, 35 responders: 81%; median duration: 18 days), third (n = 28, 20 responders: 71%; median duration: 25 days), and fourth (n = 14, 10 responders: 71%; median duration: 23 days) injections.

## Conclusion

GONB is an efficacious and reproducible treatment in CCH patients. Given the good tolerability profile when performed every 3 monthly, GONBs can play a useful role in the management of CCH, allowing frequent periods of relief from an otherwise highly disabling disorder.

